# Pneumococcal Meningitis Complicated by Cerebral Vasculitis, Abscess, Hydrocephalus, and Hearing Loss

**DOI:** 10.1155/2018/8528023

**Published:** 2018-10-16

**Authors:** Abdul Razzakh Poil, Adila Shaukat, Devendra Kumar

**Affiliations:** ^1^Consultant, Medicine, Hamad Medical Corporation, Doha, Qatar; ^2^Consultant, Radiology, Hamad Medical Corporation, Doha, Qatar

## Abstract

Intracranial abscesses, postinfectious vasculitis, and hydrocephalus are rare complications of *Streptococcus pneumoniae* (*S. pneumoniae*) meningitis, and to our knowledge, there have been no case reports where all these 3 complications occurred in a single patient with *Streptococcus pneumoniae* meningitis. Here, we report a case of a 48-year-old male who developed postinfectious vasculitis, abscess, hydrocephalus, and hearing loss after *S. pneumoniae* meningitis. Clinicians ought to be aware of the possible adverse outcomes of *S. pneumoniae* meningitis and the limitations of current treatment options.

## 1. Introduction

The mortality rate among patients with bacterial meningitis and the frequency of neurological sequelae among those who survive are high, despite major progress in intensive care and effective antimicrobial chemotherapy. Postinfectious vasculitis leading to ischemic brain damage is rare, but a known complication of bacterial meningitis and its treatment are uncertain [1–3]. The mortality rate is higher among patients with pneumococcal meningitis than among those with meningococcal meningitis [4]. This increased mortality and morbidity are due to high neurological (74.7%) and systemic (37.9%) complications. Seizure (27.6%), diffuse brain swelling (28.7%), hydrocephalus (16.1%), hearing loss (19.7%), and ischemic or hemorrhagic brain damage (21.8%) are some of the complications [5].

*Streptococcus pneumoniae* intracranial abscess is a rare complication of pneumococcal meningitis, with the majority of cases being reported in the preantibiotic era. Today, the survival rate and long-term outcome of these patients remain quite poor, with up to 35% mortality and 40% experiencing prolonged neurological deficits [6–8]. Intracranial abscesses, postinfectious vasculitis, and hydrocephalus are rare complications of *S. pneumoniae* meningitis, and to our knowledge, there have been no case reports where all these 3 complications occurred in a single patient with *Streptococcus pneumoniae* meningitis. There was a case report of postinfectious vasculitis and abscess of brain following *Streptococcus pneumoniae* meningitis [6]. Here, we report a case of a 48-year-old male with *S. pneumoniae* meningitis complicated by postinfectious cerebral vasculitis, intracranial abscess, hydrocephalus, hearing loss, and brain infarct.

## 2. Case Report

A 48-year-old Indian male with no chronic medical illness in the past admitted to emergency department with history of fever, headache, dry cough, and generalized body pain for 4 days and vomiting for one day. No abdominal pain, SOB, chest pain, joint pain, skin rash, recent travel, or exposure to sick person and no significant family history were reported. Patient denied alcohol consumption or tobacco smoking. On physical examination, the patient was well built and nourished; he was icteric, conscious, and oriented to time, place, and person. Vitals were as follows: temperature: afebrile, 35.9°C; heart rate: 94/minute; respiratory rate: 20/minute; blood pressure: 121/81 mmHg; and SpO_2_: 98% in room air.

**Figure 1:**
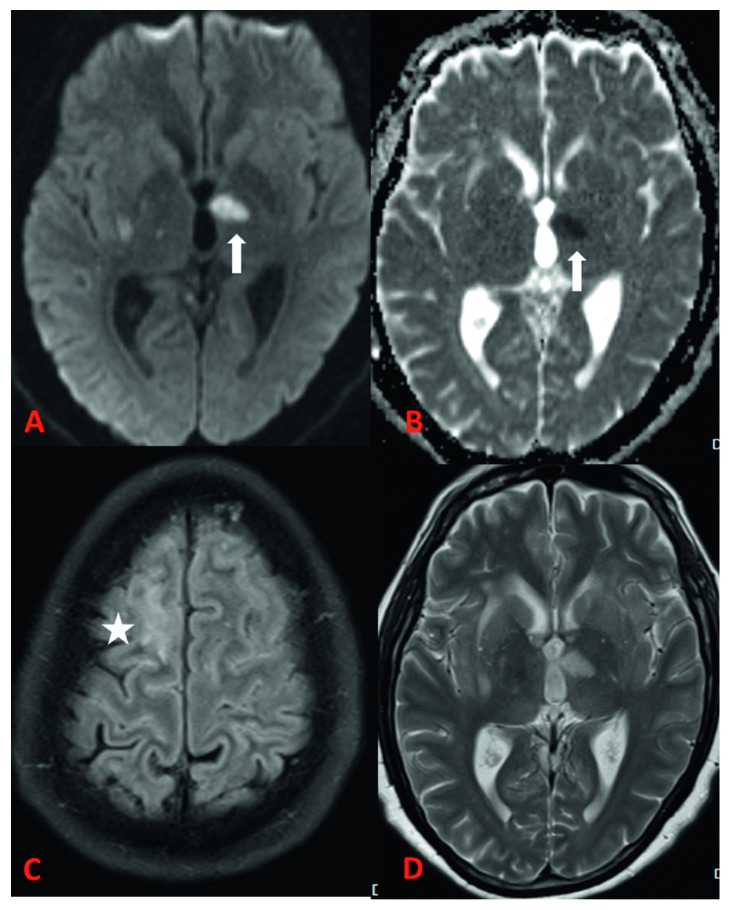
MRI brain examination of a 48-year-old male with meningoencephalitis, vasculitis, and extradural fluid collection. Axial DWI (A) and ADC (B) images show acute left thalamic (white arrow) and tiny right basal ganglia lacunar infarcts secondary to vasculitis. Axial FLAIR image (C) shows a cortical high signal in bilateral higher parietal lobes suggestive of epidural collection and underlying cerebritis (star). Axial T2-weighted image (D) shows a hyperintense signal in the left thalamus and basal ganglia secondary to vasculitis.

**Figure 2:**
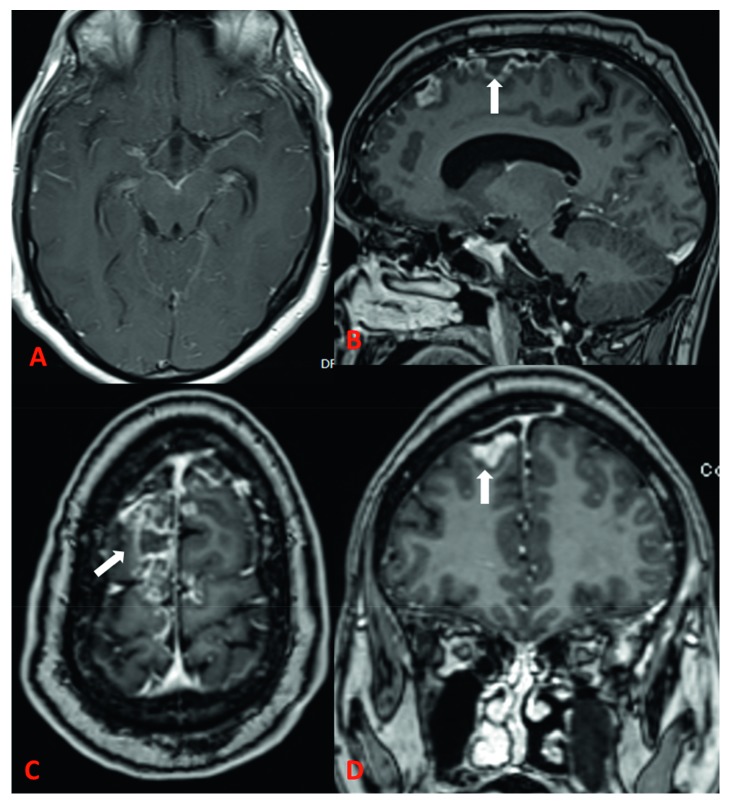
MRI brain examination of a 48-year-old male with meningoencephalitis, vasculitis, and extradural fluid collection. Postcontrast MRI axial T1 (A), GRE sagittal (B), GRE axial (C), and GRE coronal (D) images show diffuse leptomeningeal enhancement, enhancing basal exudates and high biparietal epidural fluid collection (white arrow) with cerebritis.

**Figure 3:**
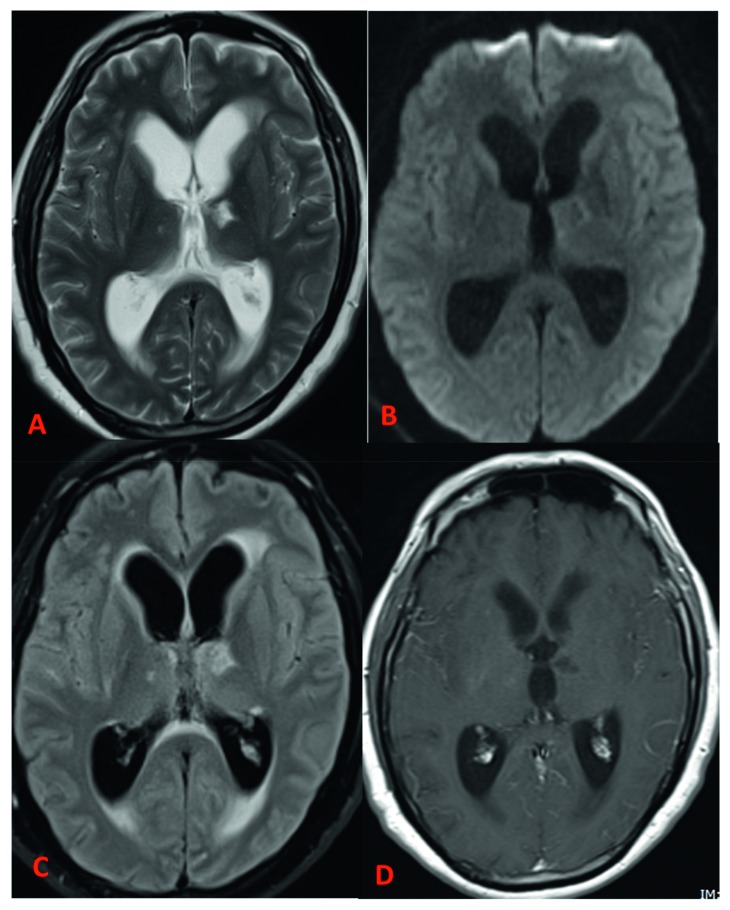
Follow-up MRI brain examination after one month. Axial T2 (A), DWI (B), FLAIR (C), and postcontrast T1-weighted (D) images show significant interval improved leptomeningeal enhancement, resolution of epidural collection (not shown), and interval new communicating hydrocephalus.

Systemic examination showed normal neurological findings except meningeal signs. Other systems were unremarkable. Initial investigations showed hemoglobin and platelets were normal. White blood cell (WBC) count was 12.6 × 10^3^/microliter (normal: 4 × 10^3^/microliter–10 × 10^3^/microliter) with 92% neutrophils. Serum creatinine was 146 *µ*mol per liter (normal: 64 to 110 *µ*mol per liter), urea was 11 mmol per liter (normal: 3.2 mmol per liter to 7.4 mmol per liter), and serum electrolytes were normal. Alanine aminotransferase (ALT) was 56 units per liter (normal: 0 units per liter to 30 units per liter), aspartate aminotransferase (AST) was 38 units per liter (normal: 0 units per liter to 31 units per liter), alkaline phosphatase (ALP) was 96 units per liter (normal: 40 units per liter to 150 units per liter), albumin was 33 g per liter (normal: 35 g/L to 50 g/L), total bilirubin was 68 *µ*mol per liter (normal: 3.4 to 20.5 *µ*mol per liter), direct bilirubin was 34 *µ*mol per liter (normal: 0 to 8.6 *µ*mol per liter), C-reactive protein (CRP) was 495 mg per liter (normal: 0 to 5 mg/L), procalcitonin (PCTN) was 11 ng per milliliter (normal: 0 to 2 ng/mL), and chest X-ray was normal. His conscious level deteriorated soon after hospital admission, and the Glasgow Coma Scale (GCS) dropped from 15/15 to 12/15. Meningitis is suspected, and antibiotics were started after lumbar puncture (LP) and computerized tomography (CT) head. Initial empirical intravenous (IV) antibiotics were started: ceftriaxone, vancomycin, and acyclovir along with dexamethasone. CT head was normal, and cerebrospinal fluid (CSF) analysis showed WBC 145 per microliter (normal: 0 to 5/*µ*L) with 96% neutrophils, protein 4.55 grams per liter (normal: 0.15 to 0.45 g/L), and Glu <0.3 mmol per liter; CSF viral panel showed Epstein–Barr virus (EBV) PCR was 326 International Units per mL, acid-fast bacilli staining was negative, and tuberculosis PCR was negative. Both CSF and blood culture showed *Streptococcus* pneumonia which was sensitive to ceftriaxone. So acyclovir and vancomycin were stopped, dexamethasone was given for a total of 3 days, and IV ceftriaxone 2 g every 12 hours was continued; later, the patient's condition improved and oriented to person but not oriented to time and place. There was no focal neurological deficit. However, he developed hard of hearing and though fever pattern has improved, having fever spike on and off. Repeat blood cultures were negative, CRP improved from >500 mg/L to 93 mg/L, PCTN decreased from 11 ng/mL to 0.41 ng/mL, and serum creatinine level normalized. However, liver enzymes persisted to rise (both transaminases and alkaline phosphatase), and white blood cell persisted to be in the range of 13–15. Due to presence of persistence of symptoms, magnetic resonance image (MRI) brain and repeat LP were done to rule out any complication of the disease. MRI brain (Figures 1 and 2) showed meningoencephalitis, vasculitis, and extradural fluid collection. There was right-sided fluid in the mastoid cavity without bone destruction. ENT was consulted and advised for medical management. As the patient is confused, hearing assessment could not be done properly. Repeat LP showed CSF WBC 11/*µ*L, neutrophils 70%, lymphocytes 29%, protein 1.11 g/L, EBV PCR was negative, and CSF culture was negative. Dexamethasone was restarted with continuation of IV ceftriaxone for a total of 6 weeks as *Streptococcus pneumoniae* meningitis is complicated by infective vasculitis, mastoiditis, and subdural collection. Repeat MRI brain (Figure 3) showed significant improvement in leptomeningeal enhancement and resolution of epidural collection; however, there was a new communicating hydrocephalus. After completion of his IV ceftriaxone, the patient was repatriated to his home country. Although his condition improved on above treatment, he was discharged with mild disorientation to time and person.

## 3. Discussion

This complex case highlights the risk of intracranial complications in invasive pneumococcal infections. Invasive pneumococcal disease is defined as an infection caused by *Streptococcus pneumoniae* isolated from a normally sterile site such as blood, cerebrospinal fluid, and pleural, joint, or peritoneal fluid. The incidence of invasive pneumococcal disease in the USA was 36.4 cases per 100,000 population above the age of 65 years, 34.2 cases per 100,000 population in infants below 1 year, and 3.8 cases per 100,000 population in the age between 18 and 34 years [9]. Data about the prevalence of invasive pneumococcal disease in Qatar are lacking. According to one report, the incidence of the disease in the general Arab region was 7% above the age of 60 years, and this is expected to increase 19% by 2050; however, incidents in Qatar population are high than those in the general Arab population (20.7%) which are comparable to those in population in the USA (26.9%) [10]. Since extremes of ages are predisposing conditions towards invasive pneumococcal disease, an effective vaccination program is very important to decrease the morbidity and mortality due to this condition. Despite effective antibiotic treatment, mortality and morbidity are high in patients with bacterial meningitis. Morbidity like vasculitis after bacterial meningitis which can lead to brain injury is one of the known complications, but its treatment is unclear [1–3]. Among bacterial meningitis, the most common organisms were *Streptococcus pneumoniae* and *Neisseria meningitidi*s. The mortality rate was significantly higher with pneumococcal meningitis (30%) when compared with meningococcal meningitis (7%) [4]. *S. pneumoniae* causes alteration in the host inflammatory response and results in the release of proinflammatory cytokines in the cerebrospinal fluid (CSF) which results in disruption of blood-brain barrier (BBB) [11]. This results in increased neurological (74.7%) and systemic (37.9%) complications. These include seizure (27.6%), diffuse brain swelling (28.7%), hydrocephalus (16.1%), hearing loss (19.7%), and ischemic or hemorrhagic brain damage (21.8%) [5]. However, vasculitis is not a common sequela of meningitis caused by *S. pneumoniae* [5]. Despite advancement in the medical field, only dexamethasone has shown to be effective adjunctive therapy. It lowers the rate of hearing loss and mortality [12]. Pneumococcal meningitis is associated with 54% of hearing loss (69% in adults and 31% in children). Advanced age, presence of comorbidity, severity of meningitis, a low CSF glucose level, and a high CSF protein level are the risk factors for hearing loss [13].

Although intracranial abscess after *Streptococcus pneumoniae* meningitis is a rare complication, the survival rate and long-term outcome are very poor, with up to 35% mortality and 40% long-term neurological sequelae [6–8].

Risk of complications in bacterial meningitis is higher in patients with advanced age, presence of otitis or sinusitis, absence of rash, a low score on the Glasgow Coma Scale, tachycardia, a positive blood culture, an elevated erythrocyte sedimentation rate, thrombocytopenia, and a low cerebrospinal fluid white-cell count [4]. Our patient has a higher risk of invasive *S. pneumoniae*. The majority of patients have an obvious secondary cause such as ear or sinus infections or pneumonia [5, 6].

Several studies have reported that hydrocephalus may occur in adults as well as in children with bacterial meningitis. The most common causative agent is *S. pneumoniae*. Among adults, the reported incidence of this complication ranged from 3 to 21%. In spite of surgical intervention, prognosis is poor with the mortality rate up to 50% [14–16].

Given the rarity of postmeningitis complications like cerebral vasculitis, sensorineural hearing loss, and hydrocephalus, there is no consensus on treatment for these conditions. It has been shown that treatment with dexamethasone in patients with pneumococcal meningitis can decrease the complication, including postinfectious vasculitis and mortality [12]. Corticosteroids have been recommended as adjunctive therapy for bacterial meningitis to reduce the complications like hearing loss especially when given early during the disease course [13, 17]. Although treatment is usually given less than four days, some reports have shown a rebound vascular inflammation after steroid withdrawal [18]. Dexamethasone therapy was shown to decrease the incidence of hydrocephalus [16]. Due to these growing bodies of the literature, we have treated our patient with a prolonged taper of steroids.

This patient's immunization status was not known; however, his immune system seems normal. He was not on any immunosuppressive medications, and he had no chronic medical illness. He did not have leukopenia, and his HIV test was negative. Hepatitis C antibody, hepatitis B surface antibody, and hepatitis B surface antigen were negative. Invasive pneumococcal disease is a vaccine-preventable disease. Currently, there are 2 vaccines available: conjugate vaccine (PCV13) and polysaccharide vaccine PPSV23 [19]. Since the introduction of conjugate vaccine in children, a gradual decrease has been noticed in the incidence of invasive pneumococcal disease both in vaccinated and nonvaccinated population most likely due to herd immunity [20–23]. But it has also been observed that pneumococcal infections due to pneumococcal serotypes that are not included in the vaccine have been rising. As per current recommendation, a dose of PCV13 has to be given followed by a dose of PPSV23 in all adults aged ≥65 years who did not receive pneumococcal vaccine before and in persons aged ≥2 years who are at high risk for pneumococcal disease like immunocompromised patients, functional or anatomic asplenia, cochlear implants, or cerebrospinal fluid leaks [24]. In Qatar, in healthy adults over 50 years, a single dose of PCV13 is given 5 years from last PPV23; additionally, high-risk adults aged 19–64 years are administered a single dose of PCV13 at a yearly interval from the last dose of PPV23, and adults who have not been previously vaccinated are administered a single dose of PCV13, followed by PPV23 eight weeks later [25].
